# Relics of interspecific hybridization retained in the genome of a drought-adapted peanut cultivar

**DOI:** 10.1093/g3journal/jkae208

**Published:** 2024-09-01

**Authors:** Paul P Grabowski, Phat Dang, Jerry J Jenkins, Avinash Sreedasyam, Jenell Webber, Marshall Lamb, Qiong Zhang, Alvaro Sanz-Saez, Yucheng Feng, Victoria Bunting, Jayson Talag, Josh Clevenger, Peggy Ozias-Akins, C Corley Holbrook, Ye Chu, Jane Grimwood, Jeremy Schmutz, Charles Chen, John T Lovell

**Affiliations:** Genome Sequencing Center, HudsonAlpha Institute for Biotechnology, 601 Genome Way Northwest, Huntsville, AL 35806, USA; National Peanut Research Laboratory, USDA-ARS, 1011 Forrester Dr SE, Dawson, GA 39842, USA; Genome Sequencing Center, HudsonAlpha Institute for Biotechnology, 601 Genome Way Northwest, Huntsville, AL 35806, USA; Genome Sequencing Center, HudsonAlpha Institute for Biotechnology, 601 Genome Way Northwest, Huntsville, AL 35806, USA; Genome Sequencing Center, HudsonAlpha Institute for Biotechnology, 601 Genome Way Northwest, Huntsville, AL 35806, USA; National Peanut Research Laboratory, USDA-ARS, 1011 Forrester Dr SE, Dawson, GA 39842, USA; Department of Crop, Soil and Environmental Sciences, Auburn University College of Agriculture, 107 Comer Hall, Auburn, AL 36849, USA; Department of Crop, Soil and Environmental Sciences, Auburn University College of Agriculture, 107 Comer Hall, Auburn, AL 36849, USA; Department of Crop, Soil and Environmental Sciences, Auburn University College of Agriculture, 107 Comer Hall, Auburn, AL 36849, USA; Arizona Genomics Institute, University of Arizona, 1657 E. Helen St., Tucson, AZ 85721, USA; Arizona Genomics Institute, University of Arizona, 1657 E. Helen St., Tucson, AZ 85721, USA; Genome Sequencing Center, HudsonAlpha Institute for Biotechnology, 601 Genome Way Northwest, Huntsville, AL 35806, USA; Department of Horticulture and Institute for Plant Breeding, Genetics & Genomics, University of Georgia, College of Agricultural and Environmental Sciences, 2360 Rainwater Road, Tifton, GA 31793-5766, USA; Crop Genetics and Breeding Research Unit, USDA-ARS, 115 Coastal Way, P.O. Box 748, Tifton, GA 31793, USA; Department of Horticulture and Institute for Plant Breeding, Genetics & Genomics, University of Georgia, College of Agricultural and Environmental Sciences, 2360 Rainwater Road, Tifton, GA 31793-5766, USA; Genome Sequencing Center, HudsonAlpha Institute for Biotechnology, 601 Genome Way Northwest, Huntsville, AL 35806, USA; Genome Sequencing Center, HudsonAlpha Institute for Biotechnology, 601 Genome Way Northwest, Huntsville, AL 35806, USA; Department of Energy Joint Genome Institute, Lawrence Berkeley National Laboratories, Mail Stop: 91R183, Berkeley, CA 94720, USA; Department of Crop, Soil and Environmental Sciences, Auburn University College of Agriculture, 107 Comer Hall, Auburn, AL 36849, USA; Genome Sequencing Center, HudsonAlpha Institute for Biotechnology, 601 Genome Way Northwest, Huntsville, AL 35806, USA; Department of Energy Joint Genome Institute, Lawrence Berkeley National Laboratories, Mail Stop: 91R183, Berkeley, CA 94720, USA

**Keywords:** peanut, *Arachis hypogaea*, drought, introgression, *Arachis cardenasii*

## Abstract

Peanut (*Arachis hypogaea* L.) is a globally important oil and food crop frequently grown in arid, semi-arid, or dryland environments. Improving drought tolerance is a key goal for peanut crop improvement efforts. Here, we present the genome assembly and gene model annotation for “Line8,” a peanut genotype bred from drought-tolerant cultivars. Our assembly and annotation are the most contiguous and complete peanut genome resources currently available. The high contiguity of the Line8 assembly allowed us to explore structural variation both between peanut genotypes and subgenomes. We detect several large inversions between Line8 and other peanut genome assemblies, and there is a trend for the inversions between more genetically diverged genotypes to have higher gene content. We also relate patterns of subgenome exchange to structural variation between Line8 homeologous chromosomes. Unexpectedly, we discover that Line8 harbors an introgression from *A.cardenasii*, a diploid peanut relative and important donor of disease resistance alleles to peanut breeding populations. The fully resolved sequences of both haplotypes in this introgression provide the first in situ characterization of *A.cardenasii* candidate alleles that can be leveraged for future targeted improvement efforts. The completeness of our genome will support peanut biotechnology and broader research into the evolution of hybridization and polyploidy.

## Teaser

The genome sequence for the drought tolerant peanut variety "Line8" is the most complete peanut genome resource available. The Line8 genome allows for improved evaluation of genome variation among peanut genotypes. Using the Line8 genome, patterns emerge relating chromosomal structural differences and genetic exchange between chromosomes. Line8 contains an introgression from a wild relative of peanut that is an important donor of disease resistance alleles, providing an additional resource for peanut crop improvement efforts.

## Introduction

Peanut (*Arachis hypogaea* L.) is an important source of oil and nutritious food consisting of proteins, vitamins, and micro-elements important in human nutrition (Arya *et al*. 2016; Moharana *et al*. 2020; Çiftçi and Suna 2022). It is mainly grown in warm climates around the world with a total production of approximately 50 M metric tons, with the United States as the 4th highest peanut producer at 2.5 M metric tons (U.S. Department of Agriculture Foreign Agricultural Service 2023). Peanut is often grown in arid, semi-arid, and dryland environments where drought stress can cause substantial productivity and economic losses. An increasing number of years of drought events threatens agriculture production and compromises food security. As a result, The Peanut Research Foundation (PRF) identified drought research as a high priority along with disease resistance, flavor and other quality traits, and aflatoxin in 2022.

Cultivated peanut is an allotetraploid (2*n* = 4*x* = 40; AABB genome) and thought to be derived from two diploid interspecific crosses between *Arachis duranensis* (AA)*×Arachis ipaensis* (BB) < 10,000 years ago (Bertioli *et al*. 2016, 2020). As a concerted goal to develop molecular breeding strategies, the genomes of the diploid progenitors, *A.duranensis* and *A. ipaensis,* were first sequenced (Bertioli *et al*. 2016), followed by the complete sequencing and annotation of cultivated allotetraploid peanut *A. hypogaea* (Bertioli *et al*. 2019; Chen *et al*. 2019; Zhuang *et al*. 2019). The development of these genomic resources spurred efforts to characterize agronomic traits for applications in molecular breeding, including genetic analysis of multiple aspects of drought tolerance (Bhogireddy *et al*. 2020; Wang *et al*. 2021, 2023).

In addition to traditional crop improvement efforts within the pure peanut gene pool, breeders have employed hybrid designs that use wild *Arachis* species as sources of resistance to biotic and abiotic stresses. An especially important example of this comes from the introgression of pest and pathogen resistance alleles from *A.cardenasii* into cultivated peanuts in the 1960s (Simpson 2001; Bertioli *et al*. 2021). However, these introgressed *A.cardenasii* alleles can negatively impact yield when the biotic stress is absent. As such, only a small set of peanut breeding lines have been selected to retain *A.cardenasii* introgressions. Despite the potentially antagonistic effects and small numbers of intentional selection events, a recent assessment of global peanut varieties found that several important peanut genotypes unexpectedly harbored *A.cardenasii* introgressions (Bertioli *et al*. 2021). The unexpectedly high frequency of these introgressions hints at either additional unknown advantages or enhanced transmission of *A.cardenasii* haplotypes.

Here, we present the genome assembly for “Line8,” a peanut line derived from two drought-tolerant genotypes (Dang *et al*. 2012, 2013). The Line8 genome assembly was undertaken both to facilitate genomic studies of the genetic and genomic underpinnings of drought tolerance and to complement the available peanut reference genomes (Bertioli *et al*. 2019; Chen *et al*. 2019; Zhuang *et al*. 2019; Newman *et al*. 2022). Taking advantage of advances in sequencing technology and bioinformatics methods, the Line8 genome is the most complete and contiguous peanut assembly to date. We use the Line8 assembly to examine structural variation between peanut genotypes, relate variation between subgenomes to tetrasomic regions and regions of genetic exchange between subgenomes, and characterize an interspecific introgression present in the Line8 genome.

## Methods and Materials

### Line8 cultivar history

Dang *et al*. (2013) evaluated five peanut genotypes challenged to early-drought stress, and “C76-16' and “Georgia Green’ had the highest drought tolerance with potentially different mechanisms based on differential gene expression (Dang *et al*. 2012). The progeny of F5 from the cross was evaluated for drought tolerance at middle season drought stress utilizing environmentally controlled shelters. The highest yielding F6 progeny under drought, “Line8', was chosen for further evaluation. In a two-year experiment performed in environmentally controlled shelters, Line8 displayed drought tolerance due to high water use efficiency via low stomatal conductance during a mid-season drought (Zhang *et al.* 2022).

### Plant growth conditions, tissue collection, and nucleic acid extractions

Four Line8 plants were grown in a sterile soil and potting medium mixture in a greenhouse at Auburn University Plant Science Research Center, Auburn, AL, in the temperature range of 20.0 to 32.0°C. Ten grams of young leaf tissue were harvested from plants at the R5 stage, flash-frozen in liquid nitrogen, and stored at −80˚C for high molecular weight (HMW) DNA extraction. Samples of roots, leaves, pods, and reproductive tissue at different stages, along with leaf tissue from well-watered and drought conditions (Supplementary Table 6), were excised and immediately flash-frozen in liquid nitrogen for RNA extraction. The putative parents, Georgia Green, and C76-16 were grown under identical conditions, and only HMW leaf tissues were harvested.

HMW DNA from Line8 was extracted using the protocol of (Doyle and Doyle 1987) with minor modifications. Flash-frozen young leaves were ground to a fine powder in a frozen mortar with liquid nitrogen followed by extraction in 2% CTAB buffer (that included proteinase K, PVP-40, and beta-mercaptoethanol) for 30 minutes at 50°C. After centrifugation, the supernatant was extracted twice with 24:1 chloroform: isoamyl alcohol. The upper phase was transferred to a new tube and combined with 1/10th volume of 3 M Sodium acetate, followed by DNA precipitated with isopropanol. DNA precipitate was collected by centrifugation, washed with 70% ethanol, air-dried for 5–10 minutes, and dissolved thoroughly in elution buffer at room temperature followed by RNAse treatment. DNA purity was measured with Nanodrop, DNA concentration measured with Qubit HS kit (Invitrogen), and DNA size was validated by Femto Pulse System (Agilent).

RNA was extracted using the Qiagen RNeasy Plant Mini Kit (Catalog# 74904). Quality was determined by using Agilent Plant RNA 6000 Nano kit (Catalog#5067-1511), and concentration was measured by Invitrogen Qubit RNA BR Assay kit (Catalog#Q10211).

### Sequencing and library construction

The Line8 PacBio HiFi library was constructed using Circular Consensus Sequencing (CCS) mode. The DNA was sheared using a Diagenode Megaruptor 3 instrument. Libraries were constructed using SMRTbell Template Prep Kit 2.0 and tightly sized on a SAGE ELF instrument (1–18 kb) to a final library average insert size of 20 kb. The accompanying Dovetail OmniC library was built using standard protocols (Dovetail Omni-C kit Catalog #21005). Illumina libraries for Line8, Georgia Green, and C76-16 were built using standard protocols (Illumina TruSeq PCRfree Catalog #20015962).

We sequenced Line8 using a whole-genome shotgun sequencing strategy and standard sequencing protocols. Sequencing reads were collected using PACBIO and Illumina platforms. PACBIO and Illumina reads were sequenced at the HudsonAlpha Institute in Huntsville, Alabama. PACBIO reads were sequenced using the SEQUEL II platform and Illumina reads were sequenced using the Illumina NovoSeq 6000 platform. For the PACBIO sequencing of Line8, 3 SMRT cells using V2 chemistry produced a total raw sequence yield of 199.3 Gb, with a total coverage of 76.59 × (Supplementary Table 5). We also sequenced one 400 bp insert 2 × 150 Illumina fragment library (38.43x) along with one 2 × 150 HiC library (75.21x; Supplementary Table 5). Prior to assembly, Illumina fragment reads were screened for phix contamination. Reads composed of >95% simple sequence were removed. Illumina reads <50 bp after trimming for adapter and quality (*q* < 20) were removed. The final Illumina read set consists of 2,048,725,820 reads for a total high-quality basepair yield of 113.64x. We sequenced libraries for Georgia Green and C76-16 (400 bp insert, 2 × 150) to 25–30 × depth on the Illumina 6000 platform (Supplementary Table 5).

### Line8 genome assembly and construction of pseudomolecule chromosomes

The version 1.0 Line8 assembly was generated by assembling the 10,350,982 PACBIO CCS reads (76.59x, 19,070-bp average read size), using the HiFiAsm + HIC assembler (v0.16.r375; Cheng *et al*. 2022) and subsequently polished using RACON (v1.4.10, Vaser *et al*. 2017). This produced an initial assembly consisting of 2,528 scaffolds (2,528 contigs), with a contig N50 of 8.3 Mb, and a total genome size of 2,485.3 Mb.

Hi-C Illumina reads from Line8 were separately aligned to the contigs with Juicer (v1.5.6, Durand *et al*. 2016), and chromosome-scale scaffolding was performed with 3D-DNA (v.180922, Dudchenko *et al*. 2017). No misjoins were identified in the assembly, and the contigs were then oriented, ordered, and joined together into 20 chromosomes (plus an alternate copy of Arahy.09) using the HiC data. A total of 541 joins were applied to the assembly. Each chromosome join is padded with 10,000 Ns. Contigs terminating in significant telomeric sequence were identified using the (TTTAGGG)_n_ repeat, and care was taken to make sure that they were properly oriented in the production assembly. The remaining scaffolds were screened against bacterial proteins, organelle sequences, and GenBank nr and removed if found to be a contaminant. After forming the chromosomes, it was observed that some small (<20Kb) redundant sequences were present on adjacent contig ends within chromosomes. To resolve this issue, adjacent contig ends were aligned to one another using BLAT (Kent 2002), and duplicate sequences were collapsed to close the gap between them. A total of 17 adjacent contig pairs were collapsed in the assembly.

Finally, homozygous SNPs and INDELs were corrected using ∼42 × of Illumina reads (2 × 150, 400 bp insert) by aligning the reads using bwa mem (v2.2.1, Li and Durbin 2009) and identifying homozygous SNPs and INDELs with the GATK's UnifiedGenotyper tool (v3.7, McKenna *et al*. 2010). A total of 4,562 homozygous SNPs and 61,888 homozygous INDELs were corrected in our release. The final version contained 2,602.2 Mb of sequence, consisting of 546 contigs with a contig N50 of 8.3 Mb and a total of 100% of assembled bases in chromosomes (Supplementary Table 7).

Completeness of the euchromatic portion of the assembly was assessed using 67,115 primary transcripts from version 1.0 *Arachis hypogaea* (Bertioli *et al*. 2019) obtained from Phytozome (Goodstein *et al*. 2012). The aim of this analysis is to obtain a measure of completeness of the assembly, rather than a comprehensive examination of gene space. The transcripts were aligned to the assembly using BLAT (Kent 2002) and alignments ≥95% base pair identity and ≥95% coverage were retained. The screened alignments indicate that 99.38% of the transcripts aligned to our assembly.

### Line8 gene and repeat annotation

Genome annotation was accomplished using the pipeline developed by the DOE Joint Genome Institute and Phytozome. Transcript assemblies were made from a total of 655.66 million pairs of 150-bp stranded paired-end Illumina RNA-seq reads (Supplementary Table 6) using PERTRAN (described in detail by Lovell *et al*. 2018). In brief, PERTRAN conducts genome-guided transcriptome short-read assembly via GSNAP (v.2013-09-30, Wu and Nacu 2010) and builds splice alignment graphs after alignment validation, realignment, and correction. Subsequently, 336,711 transcript assemblies were constructed using PASA (v2.0.2, Haas *et al*. 2003) from RNA-seq reads. Loci were determined by EXONERATE (v.2.4.0, Slater and Birney 2005) alignments of transcript assemblies and proteins from publicly available genomes including *Arabidopsis thaliana, Glycine max, Setaria viridis, Cajanus cajan, Trifolium pratense, Lotus japonicus, Medicago truncatula, Populus trichocarpa, Vitis vinifera,* and Swiss-Prot. These alignments were accomplished against repeat-soft-masked genomes using RepeatMasker (v.open.4.1.2, Smit *et al*. 2015), repeat library from RepeatModeler (v.open.1.0.11, Smit and Hubley 2010) and RepBase (https://www.girinst.org/repbase) with up to 2,000-bp extension on both ends unless extending into another locus on the same strand. Gene models with ≥ 30% Pfam TE domains and incomplete gene models, which had low homology support without full transcriptome support, or short single exon genes (<300-bp coding DNA sequences) without protein domain or good expression were removed. The R (R Core Team 2023) package GENESPACE (v1.3.1, Lovell *et al*. 2022) was used to analyze and visualize patterns of genic and repetitive sequence across the Line8 assembly.

### Mapping and phasing introgressed sequence

To evaluate the initial Line8 Arahy.09 haplotypes, we aligned the initial Line8 scaffolds to the Tifrunner (Bertioli *et al*. 2019) and *Arachis cardenasii* (Bertioli *et al*. 2021) assemblies using the “asm5” preset in minimap2 (Li 2021). To determine breakpoints between *A.hypogaea* and *A.cardenasii* sequence, we mapped PacBio HiFi reads from C76-16 and Georgia Green, the parents of Line8, to initial scaffolds showing homology to Arahy.09, using the “map-hifi” presets in minimap2 (Li 2021), retaining only the single best placement mapping. Using the alignments of Line8 to Tifrunner and *A.cardenasii* and the results from mapping C76-16 and Georgia Green to Line8, we generated the two species-resolved haplotypes for chromosome 9: “Arahy.09” which contains peanut sequence and represents a full chromosome, and “Arahy.09_alt” which contains the sequence derived from *A.cardenasii*.

### Contig breaks and telomere coordinates

We used the seqtk (Li 2016) “gap” function to identify contig breakpoints in genome assemblies. We used the “find_contigsGapsTelos” function in GENESPACE (v1.3.1, Lovell *et al*. 2022) to identify telomere sequence coordinates in the genome assemblies used in the analysis.

### Orthogroup analysis

Gene orthogroups were inferred for Line8, Tifrunner (Bertioli *et al*. 2019), and Bailey II (Newman *et al*. 2022) using GENESPACE (v1.3.1, Lovell *et al*. 2022) using default parameters. Genes in orthogroups identified as unique to Line8 were mapped to Tifrunner and Bailey II assemblies using GMAP (v.2023-10-10, Wu and Nacu 2010).

### Whole-genome alignments of peanut assemblies

We use minimap2 (Li 2021) for alignments of whole-genome assemblies, removing any sequence not included in chromosome assemblies, and using the asm5 parameter. We filtered results to retain alignments >100 kb and with percent sequence identity > 0.85 and then used SyRI (Goel *et al*. 2019) to characterize differences between genomes, including the positions of inversions.

### Parental diversity analysis

Illumina libraries for Line8 and the parents of the Line8 cross, C76-16 and Georgia Green, were mapped to Tifrunner (Bertioli *et al*. 2019) using bwa-mem2 (v2.2.1, Vasimuddin *et al.* 2019). Duplicate reads were removed using picard “MarkDuplicates” (v.3.0.0, http://broadinstitute.github.io/picard). A VCF file of variants was generated using samtools “mpileup” (v1.17, Danecek *et al*. 2021) followed by the “mpileup2cns” function in varscan (v2.4.4, Koboldt *et al*. 2012). The effects of SNPs in Line8 were predicted using SnpEff (v5.2, Cingolani *et al*. 2012).

### Tetrasomic region analysis

Line8 subgenome A (Arahy.01-Arahy.10) and Line8 subgenome B (Arahy.11-Arahy.20) were aligned using minimap2 (Li 2021) and the “asm20” parameter. SyRI (Goel *et al*. 2019) was used to identify syntenic regions and inversions between subgenomes.

Genome assemblies for *A.duranensis* and *A.ipaensis* (Bertioli *et al*. 2016; Dash *et al*. 2016) were concatenated to generate a “peanut progenitor” assembly. The peanut progenitor assembly was aligned separately to the full Line8 assembly (Arahy.09_alt omitted), the Line8 A subgenome, and the Line8 B subgenome using the “asm10” parameter in minimap2 (Li 2021). Results were filtered to retain alignments > 10 kb, with a mapping quality score >40, and with percent sequence identity > 0.7. Filtered alignments were processed in R (R Core Team 2023) to calculate percent identity within 100 kb windows across the Line8 assembly.

## Results and Discussion

### Line8 genome assembly and annotation

We sequenced the genome of the “Line8' runner-type peanut breeding line, which was selected from a pedigree of outbred progeny of two drought-tolerant genotypes “C76-16' and “Georgia Green’ (Branch 1996). To assemble the genome, we employed a whole-genome shotgun strategy. In short, we coassembled PacBio HiFi (76.59 × coverage) and Hi-C reads (75.2X) using HiFiAsm + HIC (Cheng *et al*. 2022). Contigs were subsequently scaffolded using the JUICER (Durand *et al*. 2016) pipeline generating a main assembly totaling 2,602.2Mb that was short-read polished to remove homozygous SNPs and short indels with 38.4 × Illumina 2 × 150 reads.

**Fig. 1.**
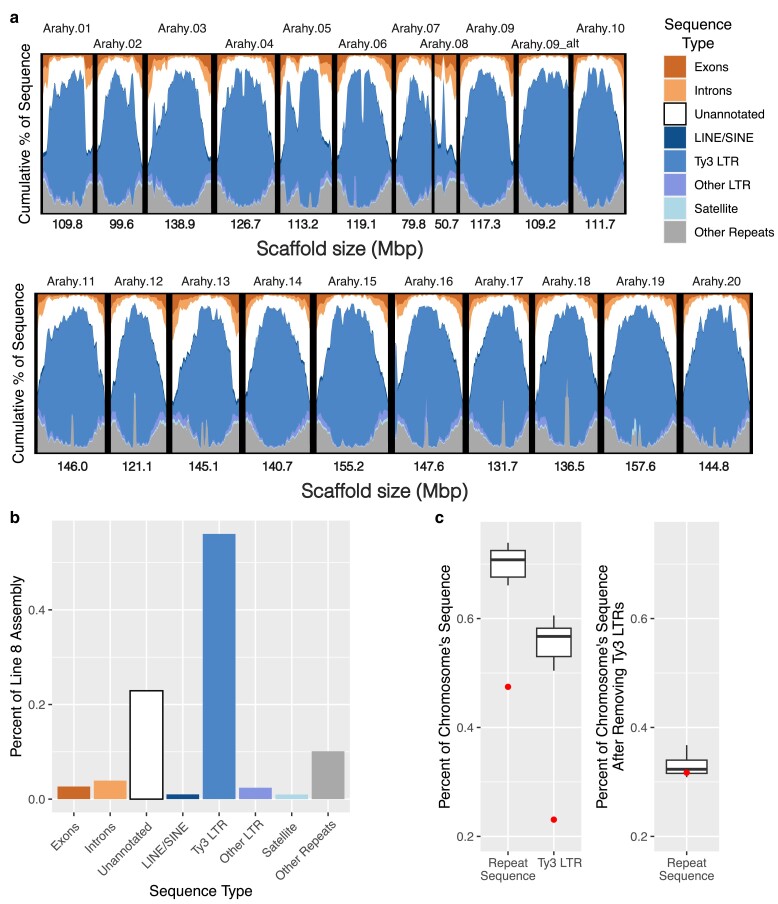
Line8 assembly and sequence content: a) the Line8 assembly consists of 21 scaffolds representing the 20 peanut chromosomes and one alternative haplotype, arahy.09_alt, representing an introgression from *Arachis cardenasii*. Plot shows the amount of sequence from exons, introns, and different types of repetitive content in 5Mb windows across each scaffold. b) Barplot showing the percent of sequence from exons, introns, and repetitive sequence in the Line8 assembly. The majority of Line8 is repetitive sequence, with Ty3 LTRs making up 56.05% of the Line8 assembly sequence. c) Boxplots showing distribution of repetitive content and Ty3 LTR sequence across the 20 Line8 chromosomes. Arahy.08 (dots) is an outlier in that it has a lower total repeat sequence and lower Ty3 LTR sequence than the other chromosomes (left panel). When Ty3 LTR sequence is removed, Arahy.08 has similar levels of repetitive sequence to the other chromosomes (right panel).

Like other peanut genotypes, the majority of the Line8 genome was inbred enough to warrant a single-haplotype representation that is typical of peanut assemblies: the Line8 genome includes a complete 2,493.0Mb representation of the 20 peanut chromosomes (Fig. 1a). However, the majority of chromosome 9 was highly heterozygous (see below) and necessitated a haplotype-resolved assembly strategy. The divergent haplotype of chromosome 9 is included in the main assembly as the 21st scaffold. Combined, the assembly has a scaffold N50 of 136.5 Mb and L50 of 9 and all scaffolds are longer than 50 Mb. The assembly is constructed from 546 contigs with contig N50 of 8.3 Mb and L50 of 93 (Table 1). The contigs contain 2,597.0 Mb and the main assembly has very little (0.2%) gap.

**Table 1.** Line8 assembly statistics.

Assembled Genome Size (Mb)	2602.2
Scaffolds	21
Contigs	546
Scaffold N50 (Mb)	136.5
Scaffold L50	9
Contig N50 (Mb)	8.3
Contig L50	94
BUSCO score	99.3

The Line8 assembly was constructed using fewer contigs (546) than any fully assembled and annotated peanut genome (Table 2) and has 100% of the unique sequence contained in its 21 chromosome-scale scaffolds. Line8 has a contig N50 (8.3 Mb) greater than other assembled genomes except the PacBio CLR-based Bailey II (Newman *et al*. 2022; Table 2). Our assembly contains identifiable telomeric sequence within at least 8 chromosome ends, with Tifrunner (Bertioli *et al*. 2019) the only available assembly with more identified telomeric regions (Table 2).

**Table 2.** Peanut assembly comparisons.

Genome Assembly	Assembly size (Mb)	Number of scaffolds	Number of Contigs	Scaffold N50 (Mb)	Contig N50 (Mb)	Gap content (%)	Genome in chromosome-scale scaffolds (%)	Number of detected telomeres	Number annotated genes	Genes in orthogroups unique to assembly	Sequencing Technology
Line8 (complete)	2602.2	21	546	136.5	8.3	0.2	100	8	55,124	6,447 (11.7%)	PacBio HiFi + HiC
Line8—20 peanut chromosomes	2,493.04	20	528	136.5	8.3	0.2	100	8	54,076	NA	PacBio HiFi + HiC
Tifrunner (Bertioli *et al*. 2019)	2556.3	384	4037	134.9	1.5	0.1	99.25	17	67,005	9,130 (13.6%)	PacBio
Fuhausheng (Chen *et al*. 2019)	2552	86	34,641	56.57	0.211	1.04	98.31	NA	83,087	NA	Illumina + PacBio
Shitouqi (Zhuang *et al*. 2019)	2538.41	20	7232	135.11	1.51	1.3	98.72	6	83,709	NA	PacBio
Kaust (Driguez *et al*. 2021)	2564	NA	285	NA	42.3	NA	NA	NA	NA	NA	PacBio HiFi
Bailey II (Newman *et al*. 2022)	2555.804	426	1004	136.83	17.57	0.22	99.29	4	62,292	9,520 (15.3%)	PacBio CLR

The Line8 assembly contains 70.5% repetitive sequence, with Ty3 LTR elements accounting for 79.5% of the repetitive sequence (Fig. 1b). Repeat content in Line8 chromosomes ranges from 66.1 to 73.9% except for Arahy.08 (47.4%, Fig. 1c) and Arahy.09_alt (79.8%, see below). The lower repeat content in Arahy.08 is primarily due to fewer Ty3 LTR elements, which account for 23.1% of Arahy.08 but 50.4–60.6% of the sequence in the other chromosomes (Fig. 1c). After Ty3 LTR elements are removed, Arahy.08 has a similar repeat content to the rest of the chromosomes (Fig. 1c).

To complement the genome assembly, we built a thoroughly supported protein-coding gene model annotation. Overall, we identified 55,124 genes in Line8, of which 37,270 (67.6%) are part of orthogroups shared with the two other peanut annotations used for comparison: Tifrunner (Bertioli *et al*. 2019) and Bailey II (Newman *et al*. 2022). The BUSCO score (Manni *et al*. 2021) for the Line8 annotation is 99.3%. Line8 contains 6,447 (11.7%) genes that are part of unique orthogroups not shared by either Tifrunner or Bailey II. The level of genes in orthogroups unique to Line8 was lower than for the other peanut annotations (Table 2) but still a surprisingly high number given the genetic similarity between peanut genotypes. However, we found that the sequences of nearly all genes in orthogroups unique to Line8 are found in the other genome assemblies with perfect or near-perfect mapping scores (Table 3), indicating that the sequence encoding almost every annotated Line8 gene is present in the other assemblies. Compared to genes from orthogroups shared by the 3 peanut annotations, genes identified as unique to Line8, on average, are shorter and have fewer exons, and a higher percentage of genes in that group map multiple times and map to non-homologous chromosomes in the other assemblies (Table 3). The discrepancies between peanut annotations, despite shared gene sequence content between assemblies, may reflect both differences in annotation methodology and differences in genome structure, and they illustrate a standing challenge in comparing gene annotations within species.

**Table 3.** Comparisons of annotations from Line8, Tifrunner (Bertioli *et al*. 2019), and Bailey II (Newman *et al*. 2022).

Line8 gene category	Number genes	Mean peptide length	Mean number of exons	% genes that map to Tifrunner/Bailey II	% genes that map multiple times in Tifrunner/Bailey II	% genes that map to non-homologous chromosome in Tifrunner/Bailey II
Genes in orthogroups unique to Line8	6447	210.7	2.86	99.7%/99.7%	39.8%/37.9%	34.1%/32.4%
Genes in orthogroups shared by Line8, Tifrunner, and Bailey II	37,270	477.5	6.29	99.9%/99.9%	23.4%/22.6%	13.6%/12.8%

### Inversions between Line8 and other peanut assemblies

One benefit of using assemblies with higher contiguity (i.e. an assembly constructed using fewer contigs) is that there are fewer opportunities for assembly errors to be spuriously interpreted as structural variation. For example, there is a high correlation between the contiguity of the Tifrunner (Bertioli *et al*. 2019), Shitouqi (Zhuang *et al*. 2019), and Bailey II (Newman *et al*. 2022) genome assemblies and the number of inversions detected between each assembly and Line8 (*r*^2^ = 0.952, *P*-value = 0.099; Table 4), suggesting a non-biological relationship between contiguity and the number of detected inversions.

**Table 4.** Inversions detected from whole-genome alignment of Line8 and Tifrunner (Bertioli *et al*. 2019), Bailey II (Newman *et al*. 2022), or Shitouqi (Chen *et al*. 2019). Filtered inversions have boundaries more than 2 kb from contig breakpoints in both assemblies used in the alignment.

Assembly Aligned to Line8	Contigs in assembly	Inversions vs Line8	Inversions within 2 kb of contig breakpoints in Line8	Inversions within 2 kb of contig breakpoints in non-Line8 assembly	Filtered inversions	Filtered inversions > 100kb	Filtered inversions > 1Mb	Number Line8 genes within filtered inversions	Genes per 1 kb across filtered inversions	SNPs vs Line8	Subspecies	Type
Tifrunner	4037	111	16 (14.4%)	79 (71.2%)	18 (16.2%)	8	3	187	0.0032	167,292	*A. hypogaea* subsp. *hypogaea*	Runner
Bailey II	1004	39	19 (48.7%)	6 (15.4%)	17 (43.6%)	10	6	286	0.0052	269,226	*A. hypogaea* subsp. *hypogaea*	Virginia
Shitouqi	7232	244	22 (9.0%)	188 (77.0%)	44 (18.0%)	25	9	530	0.0105	725,560	*A. hypogaea* subsp. *fastigiata*	Spanish

Inversions with boundaries near contig breakpoints are evidence of potential assembly errors, as misorientied contigs would appear as inversions in alignments. When an assembly has higher contiguity, there are fewer chances for potential assembly errors to show up as inversions. For example, the whole-genome alignment of Line8 (546 contigs) and Tifrunner (4037 contigs) identified 111 inversions, and 71.2% of the inversions have boundaries within 2 kb of contig breakpoints in the Tifrunner assembly compared to 14.4% for Line8 (Table 4). Inversions with boundaries near contig breakpoints could be real, as both inversions and contig breakpoints are enriched in repetitive regions (Corbett-Detig *et al*. 2019), but they could also be the result of misoriented contigs. In both Line8 and Tifrunner, regions near contig breakpoints are enriched for inversion boundaries (Line8: 198 × enrichment; Tifrunner: 453 × enrichment), but since Line8 is constructed using fewer contigs, there are fewer opportunities for both spurious inversions from assembly errors and for the boundaries of true inversions to end close to the ends of contigs. The alignment of Shitouqi (7232 contigs) to Line8 shows similar patterns, with 77.0% of the detected inversions within 2 kb of contig breakpoints in the Shitouqi assembly compared to 9.0% for the Line8 assembly (Table 4).

The benefit of higher contiguity in assemblies is further demonstrated by the alignment of Line8 to Bailey II (1004 contigs), where fewer inversions are detected relative to the comparisons of Line8 to Tifrunner or Shitouqi, but of those inversions, a much higher percentage have boundaries farther than 2 kb from contig breakpoints in either assembly and can be used with higher confidence in downstream analysis: 43.6% for the inversions between Line8 and Bailey II compared to 16.2 and 18.0% of the inversions between Line8 and Tifrunner or Shitouqi (Table 4).

After filtering, we detect 18, 17, and 44 inversions between Line8 and Tifrunner, Bailey II, and Shitouqi, respectively (Table 4). Inversions range in size from 8 kb to 43Mb. The number of genes within inversions and the gene density across inversions (i.e. genes per kb of inversion sequence) is lowest between Line8 and Tifrunner and highest between Line8 and Shitouqi, reflecting the patterns of genetic similarity between Line8 and the other assemblies (Table 4). None of the genes in any of the inversions have predicted drought response functions, but 15 genes have predicted disease resistance functions, including a set of 10 closely related genes within a 1.6Mb inversion between Tifrunner and Shitouqi on chromosome Arahy.12 (Supplementary Table 1).

### Introgression originating from unexpected outcrossing

**Fig. 2.**
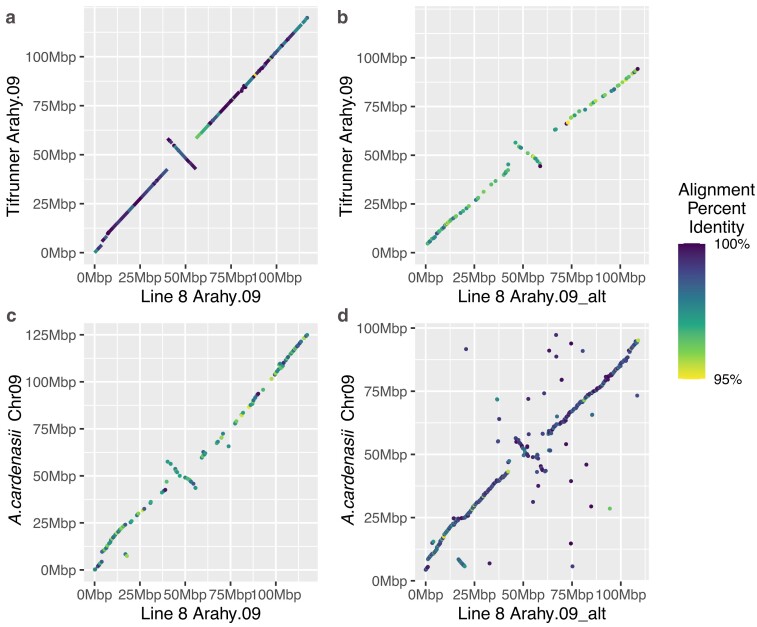
Chromosome 9 haplotype alignments show *A.cardenasii* introgression. a) Line8 Arahy.09 represents *A.hypogaea* chromosome 9 as demonstrated by its alignment with Tifrunner Arahy.09 that is complete and with high sequence similarity. b) Line8 Arahy.09_alt is highly diverged from *A.hypogaea* chromosome 9. Alignment of Line8 Arahy.09_alt with Tifrunner Arahy.09 shows gaps and lower sequence similarity compared to (a). c) Alignment of Line8 Arahy.09 with *A.cardenasii* Chr09 shows gaps and low sequence similarity, similar to alignment of Arahy.09_alt to Tifrunner Arahy.09 in (b). d) Line8 Arahy.09_alt contains sequence originating from *A.cardenasii* Chr09. Alignment of Arahy.09_alt and *A.cardenasii* Chr09 is more complete and with higher sequence similarity than (b).

The initial Line8 assembly produced contigs that generally covered each of the 20 peanut chromosomes in at single copy. However, we also produced additional contigs with homology to Arahy.09 but with unexpectedly high sequence divergence from peanut. In some peanut lines, Arahy.09 is known to harbor introgressions from *Arachis cardenasii* (Bertioli *et al*. 2021), so we compared the diverged contigs to *A.cardenasii* (Dash *et al*. 2016; Bertioli *et al*. 2021) and found that they have high similarity with *A.cardenasii* Chr09. As a result, Line8 contains two species-resolved haplotypes for Arahy.09: “Arahy.09” contains peanut-derived sequence and represents a full chromosome, and “Arahy.09_alt” contains the *A.cardenasii*-derived sequence (Fig. 2, Table 5).

**Table 5.** Results from aligning Line8 Arahy.09 haplotypes to Tifrunner Arahy.09 and *Arachis cardenasii* Chr09 (Bertioli *et al*. 2021). Statistics based on filtered alignments >10 kb and sequence identity > 85%.

Line8 scaffold	Scaffold length (bp)	% Sequence Aligned to Tifrunner Arahy.09	Sequence Identity in regions aligned with Tifrunner Arahy.09	% Sequence Aligned with *A.cardenasii* Chr09	Sequence Identity in regions aligned with *A.cardenasii* Chr09
Arahy.09	117,296,383	98.18%	98.82%	6.95%	95.72%
Arahy.09_alt	109,202,497	5.34%	95.76%	48.71%	98.26%

Line8 Arahy.09_alt contains 109Mb of *A.cardenasii*-derived sequence and aligns to a 90Mb region of Tifrunner Arahy.09 (Fig. 2). The *A.cardenassi* Chr09 introgression was previously mapped to an approximately 100Mb-110Mb region of peanut Arahy.09 (Clevenger *et al*. 2017; Bertioli *et al*. 2021), so Line8 Arahy.09_alt may not represent the full *A.cardenasii* Chr09 introgression present in some peanut breeding populations.

Arahy.09_alt has higher repeat content than the Line8 chromosomes, containing 82.1% repetitive sequence compared to 73.1% repetitive sequence in Arahy.09. This difference in repetitive content, though, is largely because the *A.cardenasii* introgression does not include the gene-rich chromosome ends (Fig. 2). As a result, Arahy.09_alt primarily contains the chromosome regions with the highest densities of repetitive sequence, and the density of repetitive elements in those regions is similar between Arahy.09_alt and the other Line8 chromosomes (Fig. 1a).

Line8 Arahy.09_alt contains 1,048 genes, including 276 orthogroups not found in the rest of Line8, and 160 orthogroups not found in any of the compared *A.hypogaea* annotations (Supplementary Table 2). Furthermore, of the nine Line8 genes that do not map to either Tifrunner or Bailey II, 5 genes are on Arahy.09_alt (Supplementary Table 2). The *A.cardenasii* Chr09 introgression confers resistance to root-knot nematode (Bertioli *et al*. 2021), with the first 4Mb of the introgression shown to be responsible for conferring strong root-knot nematode resistance (Clevenger *et al*. 2017). The first 4Mb of Arahy.09_alt contains 111 genes, though no obvious defense genes were identified within this region. Arahy.09_alt has 54.1% of the number of genes as does the same region in Arahy.09 (1048 vs 1936).

As neither of the initial Line8 pedigree parents contains the *A.cardenasii* introgression on Arahy.09, we compared variation in Line8 and its parents, Georgia Green and C76-16, by mapping Illumina reads to the Tifrunner genome assembly (Bertioli *et al*. 2019). As expected, Line8 contains variation originating from its parents: at positions with fixed differences between its parents, Line8 is heterozygous at 39.7% of SNPs and homozygous for the reference and alternate allele at approximately equal quantities (29.1% and 31.2%, Table 6). However, Line8 also contains variation not contained in either parent genotype. Line8 has an alternate allele at 302,073 SNPs where neither parent contains the alternate allele, compared to 175,665 SNPs where Line8 and one or both parents contain the alternate allele (Table 6), indicating Line8 contains substantial variation that did originate from parents of the original cross. None of the nonsynonymous SNPs in Line8 are in genes with predicted functions related to drought tolerance.

**Table 6.** Diversity in Line8 present and absent from the parents of the cross, Georgia Green and C76-16. Line8 Arahy.09_alt causes an inflated number of SNPs assigned to Arahy.09 and Arahy.19, so SNPs on Arahy.09 and Arahy.19 are omitted from the analysis.

Single nucleotide fixed differences between parents of Line8	102,844
Line8 genotypes at parental fixed difference sites: homozygous reference allele; heterozygous; homozygous alternate allele	29,931 (29.1%); 40,816 (39.7%); 32,097 (31.2%)
Positions where Line8 and one parent contain the alternate allele	175,665
Line8 positions with non-parental alleles	323,309

Since neither parental genotypes of Line8 have the *A.cardenasii* introgression and Line8 has substantial genetic variation not found in either parent, we conclude that during the rounds of self-fertilization intended to increase homozygosity in Line8, there was an inadvertent outcrossing event with a separate peanut genotype that contained the *A.cardenasii* introgression on Arahy.09. While certainly unexpected, this outcrossing event is not unheard of. An assessment of global peanut varieties by (Bertioli *et al*. 2021) found that several important peanut genotypes that were believed to be pure *A.hypogaea* in fact contained *A.cardenasii* introgressions, sometimes the result of unrecorded and/or unintended cross-pollination by introgression-containing lines.

### Tetrasomic regions, subgenomic exchange, and inversions between homeologs

**Fig. 3.**
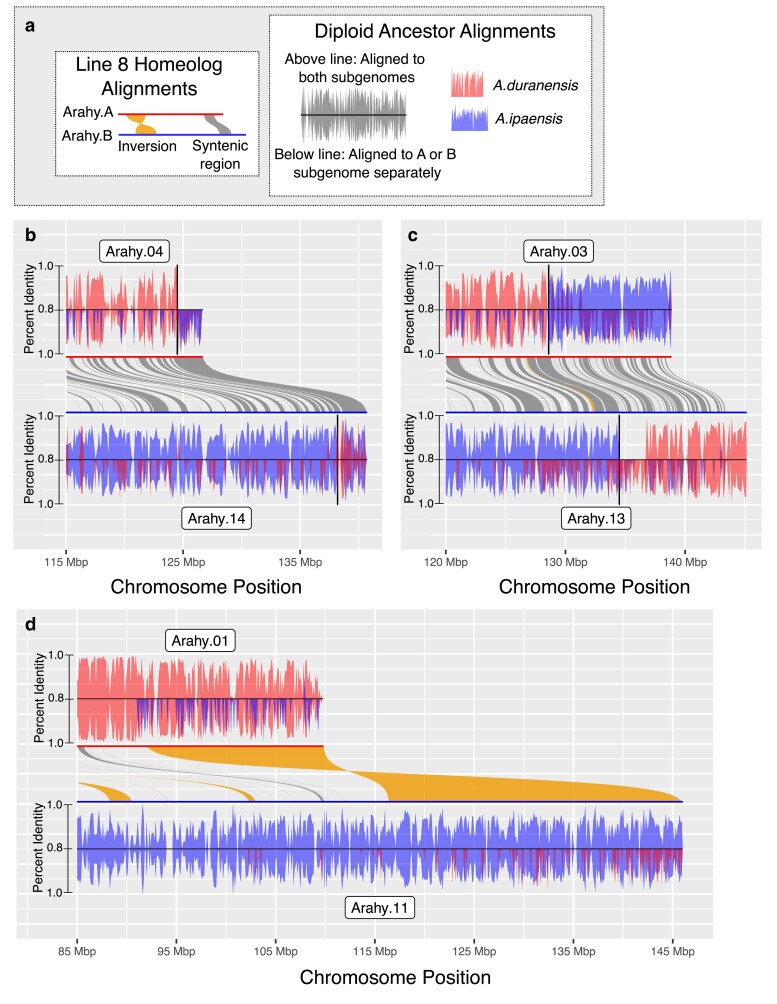
Subgenome exchange and structural variation between homeologs. a) Diagram explaining subsequent panels in the figure. *Line8 Homeolog Alignments*: Middle of each panel contains results of whole-genome alignment between Line8 homeologs, with A and B subgenome homeologs shown in red and blue, respectively. Inversions shown in orange, syntenic regions shown in dark gray. *Diploid Ancestor Alignments*: Alignments of the diploid ancestors of peanut, *A*.*duranensis* (A subgenome ancestor) and *A.ipaensis* (B subgenome ancestor) to Line8 show “A” and “B” sequence across the Line8 assembly. The top and bottom of each panel show the results for the A and B subgenome homeologs, respectively. Alignment results using all Line8 chromosomes (above line) and using only the subgenome of the homeolog (below the line) are shown. Red = *A.duranensis* alignments, blue = *A.ipaensis* alignments. Expectation is for *A.duranensis* to align best with the Line8 A subgenome and *A.ipaensis* to align best with the Line8 B subgenome. Deviations illustrate genetic exchange between peanut subgenomes. b) Alignment results for bottom of Arahy.04 and Arahy.14. Final 4Mb of both chromosomes have “B” sequence shown by better alignment of *A.ipaensis* to both regions. c) Alignment results for bottom of Arahy.03 and Arahy.13 show switch of “A” and “B” sequence in the final 10Mb of each homeolog. d) Alignment results for bottom of Arahy.01 and Arahy.11 show the quality of alignment of *A.ipaensis* to Arahy.01 (top, below line) falls dramatically near inversion boundary.

To investigate regions where there has been genetic exchange between peanut subgenomes, we aligned the reference genomes of the progenitors of the *A.hypogaea* (AABB) A and B subgenomes, *A.duranensis* (AA) and *A.ipaensis* (BB; Bertioli *et al*. 2016; Dash *et al*. 2016), to the Line8 assembly. A competitive alignment approach, where both progenitor genomes were aligned to 20 Line8 chromosomes (Arahy.09_alt omitted), identified tetrasomic regions where both progenitor genomes preferentially aligned to the same homeolog, such as the bottom of Arahy.02/Arahy.12, bottom of Arahy.04/Arahy.14, and the top of Arahy.05/15, as observed previously (Fig. 3b; Supplementary Fig. 2; Supplementary Table 3; Bertioli *et al*. 2016, 2019). We also identified regions where each ancestor preferentially aligns to the opposite homeolog, indicating a region where the sequence has swapped between homeologs, such as the bottom of Arahy.03/13 and the top of Araly.07/17 (Fig. 3c, Supplementary Fig. 2; Supplementary Table 3). The bottom of Arahy.06/16 shows both patterns, where *A.duranensis* aligns to the most distal region of both homeologs, but within the adjacent regions, each ancestor aligns with the opposite homeolog, similar to the complexity observed previously in this region (Supplementary Fig. 1; Bertioli *et al*. 2019). A subgenome-specific alignment approach, where ancestral genomes are aligned to each Line8 subgenome separately, verifies that the same ancestor aligns better to both homeologs within tetrasomic regions (Supplementary Fig. 2). The number of genes within the regions of subgenome exchange ranges from 53 to 918, with a total of 8.4% (4640) of all genes found within these regions (Supplementary Tables 3 and 4).

Genetic exchange between subgenomes results in high sequence similarity between homeologs, which created challenges for previous attempts to resolve the homeologous sequences in these regions. For example, the bottom of Arahy.02/12; the bottom of Arahy.04/14, the top of Arahy.05/15, and the bottom of Arahy.06/16 are represented by 2Mb or more of identical sequence between homeologs in the Tifrunner genome assembly (Bertioli *et al*. 2019). In the Line8 assembly, the homeologs contain unique sequences in these regions, improving our ability to examine the patterns of genetic exchange between subgenomes.

Alignment of the Line8 subgenomes shows 16 inversions greater than 5Mb between the A and B subgenomes. Inversions suppress recombination, and notably, with one exception (Supplementary Fig. 2), none of the observed tetrasomic regions contain, span, or are within inversions of any size. In the complex region at the top of Arahy.07/17, the first 700–800 kb of the chromosomes are poorly aligned except for a 150-kb inversion in the middle of the region (Supplementary Fig. 1). Within this region, competitive alignments show that the ancestors preferentially align with the alternate subgenome, but once Arahy.07 and Arahy.17 are able to be better aligned, the ancestors align to the expected subgenome.

Alignment quality of peanut ancestors to their Line8 homeologs (ex: alignment of the progenitor of subgenome A, *A.duranenesis*, to Line8 subgenome B) varied considerably across every Line8 chromosome (Supplementary Fig. 2). Ancestor-homeolog alignment quality is highest in the distal, gene-rich regions, consistent with evolutionary constraint in genic regions slowing the rate of divergence in these regions. However, disperse genetic exchange has been observed across the subgenomes (Bertioli *et al*. 2019), and those events would also decrease the divergence between Line8 chromosomes and their homeologous ancestors. Intriguingly, the boundaries of several large inversions between Line8 homeologs are near the transition in ancestor-homeolog alignment quality, such as the bottom of Arahy.01/11, the bottom of Arahy.05/15, and the top of Arahy.06/16 (Fig. 3d, Supplementary Fig. 2), suggesting that structural variation between homeologs may influence the genetic exchange between peanut subgenomes.

## Supplementary Material

jkae208_Supplementary_Data

## Data Availability

The Line8 genome assembly and annotation files are available on Phytozome (https://phytozome-next.jgi.doe.gov/) and NCBI GenBank (Assembly GCA_041383265.1; BioProject accession PRJNA1104288). All raw sequence reads have been deposited in the NCBI SRA database under BioProject accession PRJNA1104288 and PRJNA1107277; see Supplementary Table 5 for metadata and SRA information for DNA sequencing reads; see Supplementary Table 6 for metadata and SRA information for Illumina RNA-seq reads. Supplemental data files available at figshare: https://doi.org/10.25387/g3.26530861. Supplementary File 1: The percentages of sequence for each annotation within 5Mb windows across the genome. Supplementary File 2: Positions of syntenic regions and inversions between Line8 homeologs. Supplementary File 3: Percent sequence identity, in 100-kb windows, for alignments of *A.duranensis* and *A.ipaensis* to the full Line8 assembly without Arahy.09_alt. Supplementary File 4: Percent sequence identity, in 100-kb windows, for alignments of *A.duranensis* and *A.ipaensis* to Line8 subgenomes A and B separately. Supplementary File 5: VCF containing the SNPs for Line8 when mapped to the Tifrunner assembly, including predicted effects of SNPs as determined by SnpEff. Scripts used for analysis can be accessed at http://github.com/grabowsp/Peanut_Line8_assembly_analysis (DOI: 10.5281/zenodo.13376512). Supplemental material available at G3 online.
