# Supplementary material for: Relics of interspecific hybridization retained in the genome of a drought-adapted peanut cultivar
Source: G3 (Bethesda). 2024 Sep 1;14(11):jkae208. doi: 10.1093/g3journal/jkae208 (PMC11540320; doi:10.1093/g3journal/jkae208)
Supplement: jkae208_Supplementary_Data [file jkae208_supplementary_data.zip › Supplemental Material Legends.docx]

# **SUPPLEMENTAL TABLE LEGENDS**

**Table S1: Line8 Genes within Inversions**

**Table S2: Line8_Arahy.09_alt Genes in Unique Orthogroups**

**Table S3: Locations and Counts of Genes in Regions of Subgenome Exchange**

**Table S4: Line8 Genes within Regions of Subgenome Exchange**

**Table S5: DNA Sequencing Library Information**

**Table S6: RNAseq Library Information**

**Table S7: Final Line8 Assembly Summary Statistics**

# **SUPPLEMENTAL FIGURE LEGENDS**

**Figure S1: Examples of Genetic Exchange and Structural Variation Between Homeologs**

Alignments between Line8 homeologous chromosomes (middle of each panel) show syntenic regions and inversions. Alignments of peanut diploid ancestors, *A.duranensis* and *A.ipaensis*, to each Line8 homeolog (top and bottom of each panel) illustrate "A" (*A.duranensis* alignment) and "B" (*A.ipaensis* alignment) sequence. Alignments to both Line8 subgenomes (above line) and the subgenome of the homeolog (below line) are shown. **a)** Bottom of Arahy.06 (A subgenome) and Arahy.16 (B subgenome) shows that the end of each chromosome is "A" sequence, and an adjacent region where there is a sequence swap of "B" sequence on Arahy.06 and "A" sequence on Arahy.16. **b)** Complex region at top of Arahy.07 (A subgenome) and Arahy.17 (B subgenome). Beginning of chromosomes show swap of "A" and "B" sequence. After a 150kb inversion, ancestor alignments match the expected subgenome.

**Figure. S2: Genetic Exchange and Structural Variation Between Homeologs Across Line8 Assembly**

Alignments between Line8 homeologous chromosomes (middle of each panel) show syntenic regions and inversions. Alignments of peanut diploid ancestors, *A.duranensis* and *A.ipaensis*, to each Line8 homeolog (top and bottom of each panel) illustrate "A" (*A.duranensis* alignment) and "B" (*A.ipaensis* alignment) sequence. Alignments to both Line8 subgenomes (above line) and the subgenome of the homeolog (below line) are shown. Vertical orange lines show boundaries of inversions greater than 5Mb. Vertical black lines show boundaries where patterns change in the ancestor alignment results.
