# Supplementary figures and images for: Relics of interspecific hybridization retained in the genome of a drought-adapted peanut cultivar
Source: G3 (Bethesda). 2024 Sep 1;14(11):jkae208. doi: 10.1093/g3journal/jkae208 (PMC11540320; doi:10.1093/g3journal/jkae208)

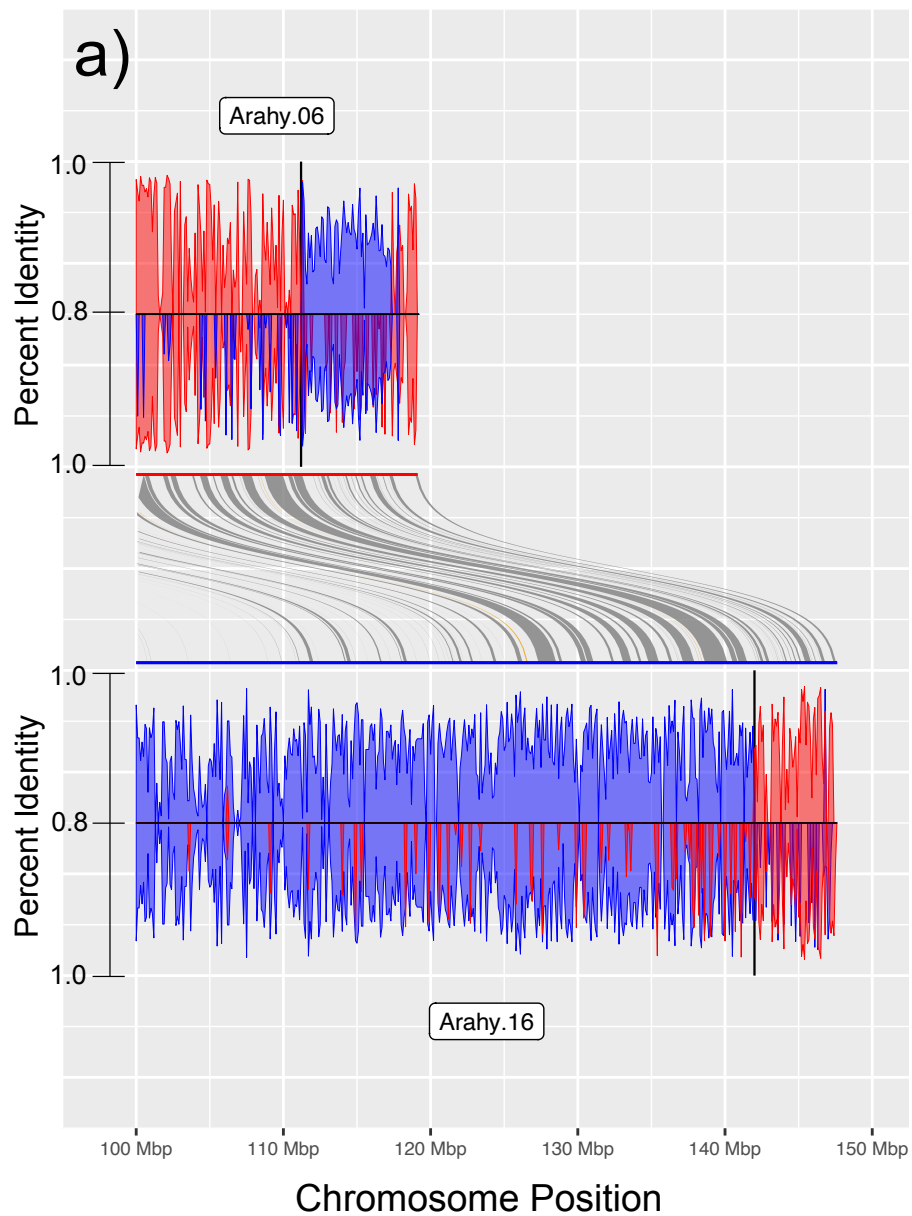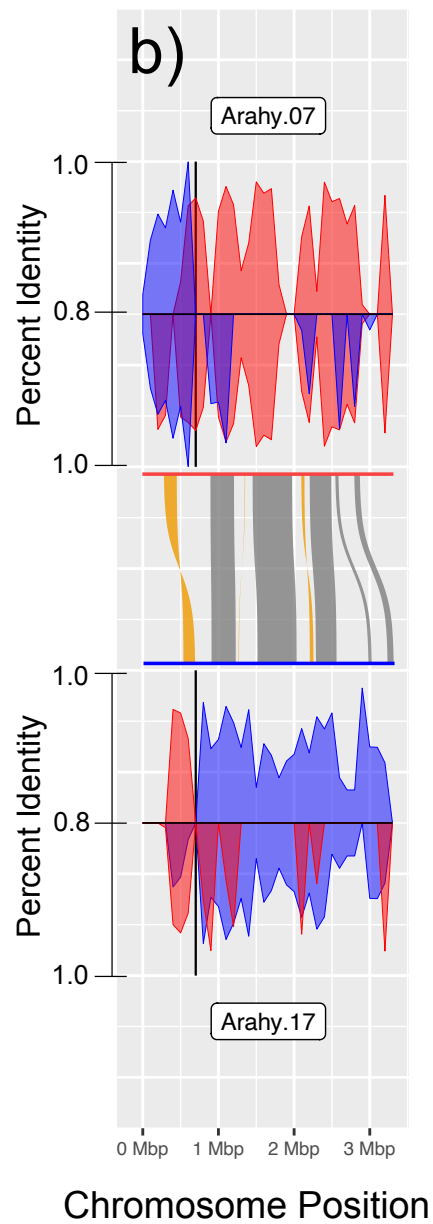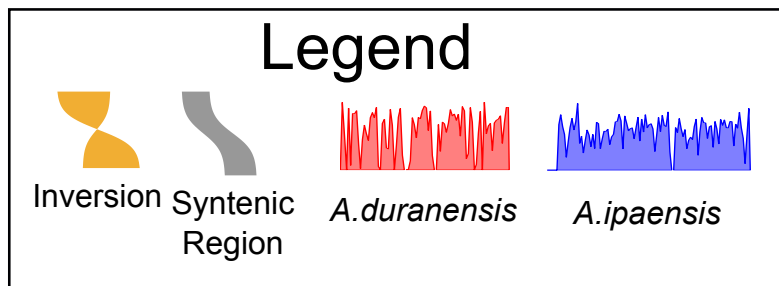

Supplement: jkae208_Supplementary_Data [file jkae208_supplementary_data.zip › Figure S1.pdf]

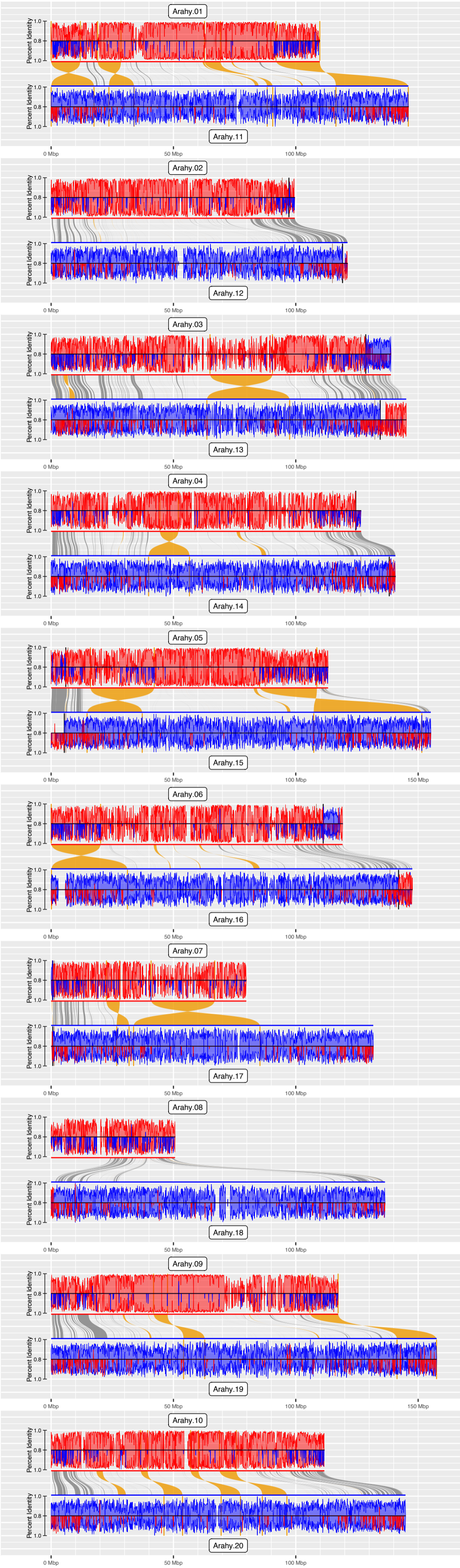

Chromosome Position

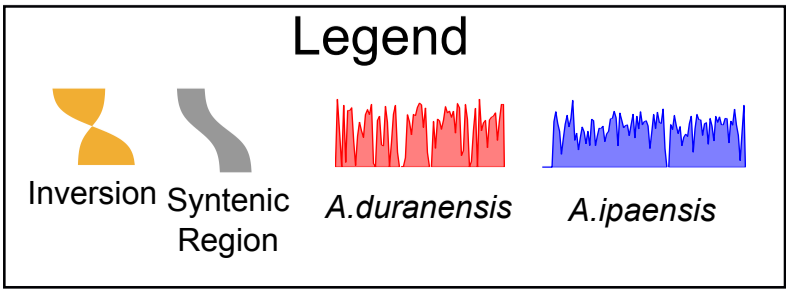

Supplement: jkae208_Supplementary_Data [file jkae208_supplementary_data.zip › Figure S2.pdf]
